# Blood or Serum Exposure Induce Global Transcriptional Changes, Altered Antigenic Profile, and Increased Cytotoxicity by Classical Bordetellae

**DOI:** 10.3389/fmicb.2018.01969

**Published:** 2018-09-07

**Authors:** Monica C. Gestal, Israel Rivera, Laura K. Howard, Kalyan K. Dewan, Illiassou Hamidou Soumana, Margaret Dedloff, Tracy L. Nicholson, Bodo Linz, Eric T. Harvill

**Affiliations:** ^1^Department of Infectious Diseases, College of Veterinary Medicine, University of Georgia, Athens, GA, United States; ^2^USDA ARS National Animal Disease Center, Ames, IA, United States

**Keywords:** *Bordetella*, inflammation, host manipulation, virulence, Blood and Serum Responsive Stimulon

## Abstract

The classical bordetellae sense and respond to a variety of environments outside and within their mammalian hosts. By causing inflammation and tissue damage, we reasoned that bordetellae are likely to encounter components of blood and/or serum during the course of a respiratory infection, and that detecting and responding to these would be advantageous. Therefore, we hypothesized that classical bordetellae have the ability to sense and respond to blood or serum. Blood or serum exposure resulted in substantial transcriptional changes in *Bordetella bronchiseptica*, including enhanced expression of many virulence-associated genes. Exposure to blood or serum additionally elicited production of multiple antigens not otherwise detectable, and led to increased bacterial cytotoxicity against macrophages. Transcriptional responses to blood/serum were observed in a Bvg^−^ phase-locked mutant, indicating that the response is not solely dependent on a functional BvgAS system. Similar transcriptional responses to blood/serum were observed for the other classical bordetellae, *Bordetella pertussis* and *Bordetella parapertussis.* These data suggest the classical bordetellae respond to signals present in blood and serum by changing their behavior in ways that likely contribute to their remarkable success, via effects on pathogenesis, persistence and/or transmission between hosts.

## Introduction

Bacteria can sense and respond to many environmental cues, such as temperature, pH, oxygen, and iron, changing their behavior to better adapt to the variables of their surroundings (Sperandio et al., 2003; Anderson and Armstrong, 2006; Pacheco and Sperandio, 2009; Parker and Sperandio, 2009; Curtis and Sperandio, 2011; Armstrong et al., 2012; Hester et al., 2012; Kendall and Sperandio, 2016). Bacteria that can be both commensals and pathogens are likely to encounter different cues associated with very different challenges, and can benefit from the ability to respond appropriately to each. The classical *Bordetella* species are recognized as both highly virulent pathogens and as symptom-free commensals, blurring the line between these categories and presenting an opportunity to examine what is necessary to be successful as either, or both. *B. pertussis* is the recognized cause of a virulent and highly contagious coughing illness, pertussis (whooping cough), but is also believed to transmit frequently amongst individuals with little or no disease (Mattoo and Cherry, 2005; Warfel et al., 2014; Kilgore et al., 2016). *Bordetella bronchiseptica* is a common commensal of the respiratory tracts of many mammals, including those of mice and men, and can also cause various mild to severe respiratory diseases. *B. bronchiseptica* naturally infects mice, and a remarkably low dose (5 CFU) is sufficient to consistently and persistently colonize the nasal cavity (Weyrich et al., 2014). Over the course of weeks, the bacteria grow in numbers and spread to the lower respiratory tract and other organs, demonstrating the ability to survive in these very different environments. As such, this experimental system offers the opportunity to examine how bacteria respond to natural physiological cues in different organs and different diseased states that could alert them to the particular challenges in those different environments.

The ability of *Bordetella* species to sense and respond to the host has been primarily studied in the context of the relatively simple BvgAS two-component system, a paradigm for a “virulence regulon”. In classical bordetellae the expression of most identified virulence-related genes is regulated by the two-component system encoded by the *bvgAS* locus. This locus encodes a histidine kinase sensor protein, BvgS, and a DNA-binding response-regulator protein, BvgA. In response to environmental cues such as temperature (37°C), BvgS activates BvgA by phosphorylation, and BvgA subsequently binds to the promoter regions of the Bvg^+^ regulated genes and activates transcription. In the Bvg^+^ phase, many of the known virulence factors are expressed, including filamentous hemagglutinin (FHA), pertactin (PRN), fimbriae (FIM), adenylate cyclase toxin (ACT), and dermonecrotic toxin (DNT), as well as a type III secretion system (TTSS) all known for their important role during different stages of the infectious process within the host. In the Bvg^−^ phase virulence factors are down-regulated and expression of other genes such as those necessary for motility and adaptation during inter-host conditions, are up-regulated. In the laboratory, bacteria in the Bvg^+^ phase can be recognized by their hemolytic phenotype on blood agar plates, which is absent in the Bvg^−^ phase (Preston et al., 2003). In addition to low temperature, modulation from Bvg^+^ to Bvg^−^ can be induced by the presence of MgSO_4_ or nicotinic acid (Scarlato and Rappuoli, 1991; Cotter and Miller, 1994; Stenson and Peppler, 2007). The relatively simple paradigm of Bvg-regulated virulence has been revealed to be an oversimplification by several studies involving other two-component systems such as RisAS (Jungnitz et al., 1998; Coote, 2001; Zimna et al., 2001; Chen et al., 2017) or PlrSR (Bone et al., 2017) which also regulate aspects of virulence. In addition, several groups have demonstrated that *Bordetella* species sense and respond to a variety of potential within-host cues including iron and CO_2_ (Brickman et al., 2011; Armstrong et al., 2012; Hester et al., 2012; Valdez et al., 2016). These observations suggest there is still much to learn about the regulation of gene expression and its role in different aspects of *Bordetella* pathogenesis.

Based on these findings, we speculated that bordetellae can respond to signals particular to sites and/or conditions such as inflammation. We reasoned that tissue damage caused during respiratory infection would be expected to expose bacteria to blood and/or serum components, and that sensing those would allow the bacteria to protect itself from a variety of associated anti-microbial factors. Therefore, we hypothesized that the classical bordetellae have the capacity to sense and respond to blood or serum components that they are likely to encounter during the course of a respiratory infection. To test this hypothesis we investigated the global transcriptional response of Classical *Bordetella* species exposed to blood or serum. We identified a Blood/Serum Responsive Stimulon (BSRS) that includes an altered antigenic profile and increased cytotoxicity. These effects, observed in wild type and Bvg mutants, reveal layers of regulation controlling virulence gene expression in response to cues particular to different locations and conditions within the host environment.

## Materials and Methods

### Bacterial Strains and Culture Conditions

The strains used in this study are listed in **Table 1**. They include *B. pertussis* strain 536 (a derivative of Tohama_I with resistance to streptomycin) (Weiss et al., 1983), *B. parapertussis* human strain 12822 (Heininger et al., 2002), *B*. *bronchiseptica* strain RB50 and its Bvg phase-locked mutants RB53 (Bvg^+^) and RB54 (Bvg^−^) (Cotter and Miller, 1994). Bacteria were grown on Bordet-Gengou agar plates (Difco) supplemented with 10% sheep blood (Hemostat, Dixon, CA, United States) or in liquid Stainer-Scholte medium (SS) (Stainer and Scholte, 1970). Sheep blood was kept at 4°C before use. Heat inactivated, defibrillated sheep serum (Sigma-Aldrich) was stored as aliquots at −20°C.

**Table 1:** Strains included in this study.

Species	Strain name	Description	Reference
*Bordetella bronchiseptica*	RB50	Wild-type	Cotter and Miller, 1994
*Bordetella bronchiseptica*	RB53	Bvg^+^ phase-locked	Cotter and Miller, 1994
*Bordetella bronchiseptica*	RB54	Bvg^−^ phase-locked	Cotter and Miller, 1994
*Bordetella pertussis*	BP536	Tohama I streptomycin resistant	Weiss et al., 1983
*Bordetella parapertussis*	BPP12822	Human isolate	Heininger et al., 2002

### RNA Isolation and cDNA Synthesis

Three independent biological replicates of *B. bronchiseptica* strains RB50 and RB54, *B. pertussis* strain BP536 and *B. parapertussis* strain BPP 12822 were grown in SS medium at 37°C with shaking (220 rpm) to the exponential growth phase at an optical density of OD_600_ = 0.7. The cultures were washed twice with PBS buffer, and equal aliquots were resuspended in SS medium (control), in 100% sheep serum, or in 100% sheep blood, and incubated for 1 h at 37°C. The experiments were performed in independent triplicates. Bacteria were harvested by centrifugation at 13,000 rpm for 3 min, and the pellet was washed twice with PBS. Total RNA was extracted from the bacteria using the RNAeasy Kit (Qiagen, Valencia, CA, United States), and treated with RNase-free DNase I (Invitrogen, Carlsbad, CA, United States) according to the manufacturer’s instructions. Approximately 3 μg of RNA was used for cDNA synthesis in a 20 μl volume. A pool of random primers and 2 μl of 10 mM deoxynucleoside triphosphates (dNTPs) were added to the mixture before denaturation at 65°C for 5 min. The mixture was placed on ice, and the first-strand buffer (500 mM KCl, 200 mM Tris-HCl [pH 8.4], 5 mM MgCl_2_), 10 mM DTT and 40 units of RNase Out (Invitrogen) were added. The reaction mixture was then incubated at room temperature for 10 min before the addition of 200 units of SuperScript III reverse transcriptase (Invitrogen), followed by incubation at 50°C for 1 h. The reactions were terminated by incubation at 85°C for 5 min. As a negative control, a mock cDNA synthesis reaction was carried out in parallel by excluding the reverse transcriptase from the mixture.

### Preparation of Labeled cDNA and Microarray Analysis

A 2-color hybridization format was used for the microarray analysis. For each biological replicate, RNA extracted from RB50 after 1 h exposure to blood or serum was used to generate Cy5-labeled cDNA and RNA extracted from RB50 after 1 h exposure to SS medium was used to generate Cy3-labeled cDNA. For microarray analysis of RB54 exposure to blood, RNA extracted from RB54 after 1 h exposure to blood was used to generate Cy5-labeled cDNA and RNA extracted from RB54 after 1 h exposure to SS medium was used to generate Cy3-labeled cDNA for each biological replicate. We chose to incubate RB54 in blood as we predict that blood will reveal the most significant differences in gene expression, because when *Bordetella* spp. is in the host environment, in normal physiological conditions, it will be in contact with blood not 100% serum. Dye-swap experiments for each condition were performed analogously, in which the fluorescent labels were exchanged to ensure that uneven incorporation did not confound our results. Fluorescently labeled cDNA copies of the total RNA pool were prepared by direct incorporation of fluorescent nucleotide analogs during a first-strand reverse transcription (RT) reaction described above, followed by buffer exchange, purification, and concentration as described (Nicholson, 2007; Buboltz et al., 2008, 2009; Nicholson et al., 2009, 2012; Hester et al., 2012; Sukumar et al., 2014). The two differentially labeled reactions to be compared were combined and hybridized to a *B. bronchiseptica* strain RB50 specific long-oligonucleotide microarray (Nicholson et al., 2012; Sukumar et al., 2014). Slides were then scanned using a GenePix 4000B microarray scanner and analyzed with GenePix Pro software (Axon Instruments, Union City, CA, United States). Spots were assessed visually to identify those of low quality and arrays were normalized so that the median of ratios across each array was equal to 1.0. Spots of low quality were identified and were filtered out prior to analysis. Ratio data from the biological replicates were compiled and normalized based on the total Cy3% intensity and Cy5% intensity to eliminate slide-to-slide variation. Gene expression data were then normalized to 16S rRNA. The statistical significance of the gene expression changes observed was assessed by using the significance analysis of microarrays (SAM) program (Willems et al., 1990). A fourfold change in expression (2 log2) was set as the cut-off. A one-class unpaired significant analysis of microarrays using a false-discovery rate of 0.001% was performed. Hierarchical clustering of microarray data using Euclidean distance metrics and average linkage clustering was performed using MeV software from TIGR (Tusher et al., 2001).

### Quantitative Real-Time PCR

Quantitative real-time PCR (qRT-PCR) analyses were performed on a QuantStudio5 (Applied Biosystems) using a PCR Master Mix with an asymmetrical cyanine dye (Applied Biosystems) and 0.5 μM of forward and reverse primer. The primer sequences were selected from the NCBI database (**Supplementary Table 1**) and purchased from Integrated DNA Technologies (IDT, Coralville, IA, United States). The thermocycler protocol was set to a 10 min pre-incubation stage at 95°C, followed by 31 cycles of a two-step PCR for a denaturing phase at 94°C for 15 s, a combined annealing and extension phase at 60°C for 60 s, and a final melt curve stage. Gene expression was calculated using the ΔΔCt method and *recA* for normalization. To determined the statistical significance a Two-sample, two-tailed Student’s *t*-test comparing the 2^−ΔCt^ values of the two groups was performed, and *p*-value was subsequently calculated. Finally, *P*-values were adjusted using the Benjamini–Hochberg FDR method (Bendor et al., 2015; Taylor-Mulneix et al., 2017).

### Coomassie Staining and Western Blot

Cultures were grown in SS medium to a late exponential phase (OD_600_ = 1), and bacteria from 1 ml were harvested by centrifugation at 13,000 rpm for 3 min. The bacterial pellet was washed twice with PBS, resuspended in 100% serum or in SS medium (control), and incubated for 1 h at 37°C. The bacteria were pelleted by centrifugation at 13,000 rpm for 2 min and re-suspended in 200 ml of 4X SDS-sample buffer supplemented with 5% β-mercaptoethanol. The samples were denatured at 95°C for 10 min, loaded onto two 4–12% Tris-glycine gels (Novex^TM^WedgeWell^TM^, Invitrogen) and run at 100 V, 500 A for 2 h. One gel was Coomassie-stained using the Pierce^TM^ Power Staining Kit (Thermo Fisher Scientific) following the manufacturer’s recommendations the other gel was blotted onto a PVDF membrane using the Pierce Power Blot Cassette following manufacturer’s protocols. The Western blot protocols were performed as described previously (Yuk et al., 1998), adding 1:5000 of primary antibody (serum obtained from a previously infected mouse) and 1:10,000 of Goat anti-Mouse IgG-HRP conjugated secondary antibody (Thermo Fisher Scientific, Invitrogen, A16072). All Western Blots were developed using the SuperSignal West Femto Maximum Sensitivity Substrate (Thermo Fisher Scientific) following manufacturer’s recommendations.

We chose to study protein expression after incubation in serum and not in blood, because the serum increases expression of virulence factors as shown in the microarray and it is easier to process. As a control we performed the same gels using only serum, to be able to detect those bands, which could be non-specific markers.

### Cytotoxicity Assay

RAW murine macrophages RAW264.7 (for *B. bronchiseptica*) or THP-1 human macrophages (for *B. pertussis* and *B. parapertussis*) were cultured in RPMI medium 1640 (Gibco, Life Technologies) supplemented with 10% Fetal Bovine Serum (FBS) (Gibco, Life Technologies) and 100 U/ml Penicillin-Streptomycin (Gibco, Life Technologies) to 90% confluency. The macrophages were washed twice with RPMI supplemented with 2% FBS as previously described (Yuk et al., 1998; Bendor et al., 2015). Exponentially growing cultures of *B. bronchiseptica*, *B. pertussis*, and *B. parapertussis* in were harvested by centrifugation, washed twice in PBS, and resuspended in SS medium or in SS medium supplemented with increasing concentrations of serum (0.5, 32, and 100%). To perform these studies we used only serum because cytotoxicity is a colorimetric reaction and blood was altering the results when measuring absorbance. The bacteria were added to the macrophages at an MOI of 10, and the samples were centrifuged at 300*g* for 10 min. The preparations were incubated for 1 h at 37°C in a 5% CO_2_ atmosphere. The cytotoxicity was measured as the release of LDH using the CytoTox 96 Kit (Promega) following the manufacturer’s protocol. This assay is specific for measuring the LDH release of eukaryotic cells, but as a control, we performed the same assay on media containing bacteria at the same concentration, but not macrophages, revealing that no-cytotoxicity is detected in bacteria alone and further confirming that the increase in LDH release was due to increase in macrophage death.

### Animal Experiments

All animal experiments were carried out in strict accordance with the recommendations in the Guide for the Care and Use of Laboratory Animals of the National Institutes of Health. The protocol was approved by the Institutional Animal Care and Use Committee at the University of Georgia, Athens, GA, United States (A2016 02-010-Y2-A3 “Bordetella–Host Interactions”). All animals were anesthetized using 5% isoflurane and euthanized using carbon dioxide inhalation followed by cervical dislocation to minimize animal suffering.

Exponentially growing cultures of *B. bronchiseptica* RB50*, B. pertussis* 536, or *B. parapertussis* 12822 in SS were adjusted to an OD_600_ of 0.1. Aliquots of 1 ml (approximately 10^8^ bacteria) were pelleted by centrifugation and subsequently incubated in either SS medium, 100% blood or 100% serum for 1 h at 37°C. The bacteria were pelleted by centrifugation at 13,000 rpm for 3 min, and the pellets were washed twice with PBS. To prepare the inoculum the bacterial pellets were resuspended in PBS to an OD_600_ = 0.1, which contains approximately 10^8^ bacteria per ml. The suspension was diluted 10-fold in PBS to 10^7^ bacteria/ml (or 5 × 10^5^ bacteria in 50 μl). Six week old female C57BL/6J mice (Jackson Laboratories, Bar Harbor, ME, United States) were intranasally inoculated by pipetting 50 μl of PBS containing 5 × 10^5^ bacteria onto the external nares. Bacterial numbers in the inoculum were validated by plating serial dilutions. Three days post-inoculation, mice were sacrificed, and respiratory organs (nasal cavity, trachea, lungs) were extracted to assess bacterial colonization. Tissues were homogenized in 1 ml of PBS, serially diluted and plated onto BG agar plates with 200 μg/ml streptomycin, and colonies were counted after 2 to 4 days of growth at 37°C.

To obtain serum from convalescent mice, mice were infected with 5 × 10^5^ bacteria as above, and blood was collected from the hearts of euthanized mice 28 days post-inoculation. The samples were centrifuged at 13,000 rpm at 4°C for 30 min. After centrifugation the supernatant (serum) was collected, transferred to a clean tube and kept at −20°C until use.

### Rigor

All experiments were performed in at least three independent replicates. To ensure reproducibility, commercially available sheep blood (Hemostat) and sheep serum (Sigma-Aldrich) were used for the experiments to diminish error due to individual sources of blood or serum dependent on specific animals.

### Microarray Data Accession Number

All microarray data are available in the **Supplementary Material** and have been deposited in Array Express under accession numbers E-MTAB-6145, E-MTAB-6146, and E-MTAB-6147.

## Results

### Defining a Blood Responsive Stimulon in *B. bronchiseptica*

**FIGURE 1:**
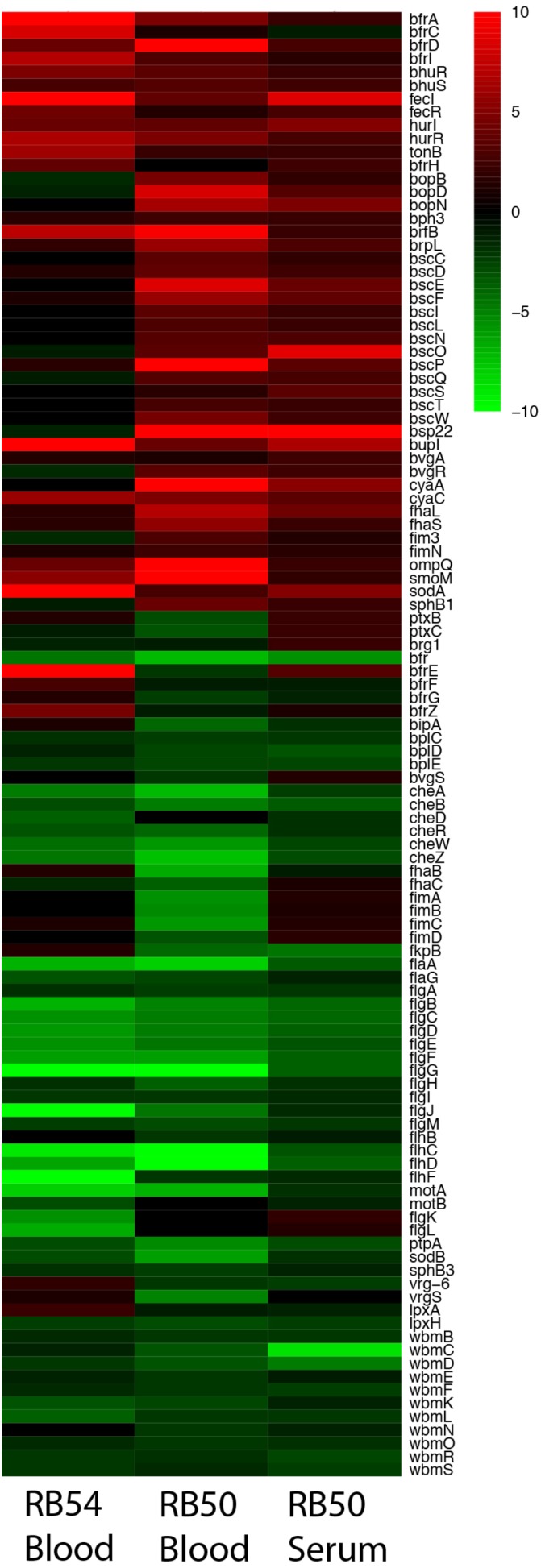
Expression profiles representing transcriptional changes of annotated *B. bronchiseptica* (RB50) genes after exposure to blood or serum. The transcriptional changes exhibited by the Bvg^−^ phase-locked mutant (RB54) following exposure to blood is shown in the left column, transcriptional changes exhibited by wild-type *B. bronchiseptica* following exposure to blood is shown in the center column, and transcriptional changes exhibited by wild-type *B. bronchiseptica* following exposure to serum is shown in the right column. Data are mean centered for each array element and averaged from three biological replicates. All expression profiles of genes are in rows with annotated gene names shown to the right and are represented using the color scale at the top right. Red – increased gene expression; green – decreased gene expression; black – no significant change in gene expression (A one-class unpaired significant analysis of microarrays using a false-discovery rate of 0.001% was performed).

Microarray analysis was performed to measure the global transcriptional response of *B. bronchiseptica* after exposure to blood or serum. Eighty-nine genes were identified as significantly up-regulated (*p* < 0.0001) after exposure to blood and eighty-eight genes were identified as significantly up-regulated (*p* < 0.0001) after exposure to serum (**Figure 1** and **Supplementary Tables 2**, **3**). Fifteen genes were commonly up-regulated upon blood or serum exposure (**Table 2**), including genes encoding the adenylate cyclase toxin/hemolysin (*cyaA*), several proteins of the type III secretion system (T3SS), an adhesin (*fhaL*), a FutA1-like iron transporter as well as putative membrane and exported proteins (**Table 2**).

**Table 2:** Genes up-regulated in blood and serum.

Locus tag	Protein
BB0324	*CyaA*
BB0457	Enoyl-CoA hydratase ortholog
BB0458	Carboxymuconolactone decarboxylase
BB1292	Membrane protein
BB1616	*BopN*
BB1617	*Bsp22*
BB1639	Type III secreted protein BspR [*Bordetella bronchiseptica* MBORD624]
BB1721	Peptidase [*Bordetella parapertussis*]
BB1936	FhaL
BB2946	FutA1-Like
BB3099	Hypothetical protein
BB3119	DoxX family protein [*Bordetella bronchiseptica* 99-R-0433]
BB3241	Hypothetical protein
BB3280	Hypothetical protein
BB3660	ECF family RNA polymerase sigma factor
BB4227	Hypothetical protein
BB4742	ECF sigma factor Previously sequenced as *Bordetella bronchiseptica* ECF sigma factor BupI

*This table shows the locus_tag of those genes that appear up-regulated on both conditions, after incubation with sheep blood and sheep serum.*

Among the genes up-regulated specifically in response to blood were type V secretion auto-transporter genes (BB3111, BB3293, and BB2033) and several iron acquisition genes. Interestingly, some iron acquisition genes were up-regulated in serum but not in blood, as was expression of many phage-related genes (**Supplementary Tables 2**, **3**). Among the genes down-regulated in response to blood were those involved in O-antigen and LPS synthesis, Type 6 secretion system (T6SS), and genes involved in flagella synthesis, function and in chemotaxis. Overall, these data indicate that there is a Blood/Serum Responsive Stimulon (BSRS) in *B. bronchiseptica* that includes genes encoding several well-characterized virulence factors.

Many of the significantly up- or down-regulated genes in either blood or serum are known to be controlled by the BvgAS system. To examine the contribution of Bvg to the blood response, a microarray analysis was performed on the Bvg^−^ phase-locked mutant, strain RB54, after exposure to blood. In the Bvg^−^ phase virulence factors were down-regulated and expression of other genes such as those necessary for motility and inter-host phase, were up-regulated. Those genes that appear up-regulated after exposure to blood in this strain, might be controlled by alternative regulatory systems. Ninety genes were identified as significantly up-regulated (*p* < 0.0001) after exposure to blood in the Bvg^−^ phase-locked mutant indicating that transcriptional activation of these genes may be Bvg-independent, including genes encoding virulence factors and proteins involved in iron acquisition such as *bfrI*, *bfrZ*, *bhuR*, *hurR*, *tonB*, and *brfD*. Importantly, known Bvg^−^regulated genes including *cyaY*, *cyaB*, *cyaD*, were up-regulated by blood in the mutant RB54 (**Figure 1**), suggesting Bvg^−^independent regulatory mechanisms. Twenty-eight genes were up-regulated in both RB54 and RB50 exposed to blood, including proteins involved in iron acquisition, putative secreted and exported proteins, and transcriptional regulators, among others. Six genes were significantly up-regulated in all three datasets: genes encoding iron-regulated membrane proteins (locus_tags BB1721, BB2946, and BB3099), RNA polymerase sigma factor BupI (BB4742), and 2 proteins of unknown function (BB3280, BB3730). This suggests that these genes play a key role in the host–pathogen interaction and because of their great importance they might have alternative mechanisms of regulation than only Bvg.

In contrast, genes encoding T3SS components were observed to be up-regulated after blood exposure by wild-type *B. bronchiseptica* RB50, but not by the Bvg^−^ phase-locked mutant, indicating that their blood responsiveness is Bvg-dependent. Collectively our data indicate that blood or serum exposure induce global transcriptional changes in *B*. *bronchiseptica*, some of which are independent of the BvgAS system.

### Serum Exposure Alters the Antigenic Profile of the Classical *Bordetella* Species

**FIGURE 2:**
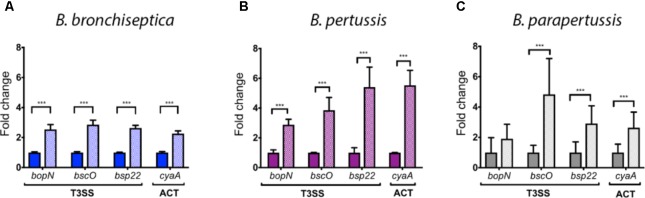
Serum-induced up-regulation of T3SS and ACT gene expression in classical *Bordetella* species as quantified by qRT-PCR. Differential expression of three genes encoding components of the T3SS (*bopN*, *bscO*, and *bsp22*) and of the *cyaA* gene (ACT) of *Bordetella* incubated in SS medium (open box) and incubated in 100% sheep serum for 1 h (hatched box). **(A)** *B. bronchiseptica*. **(B)** *B. pertussis*. **(C)** *B. parapertussis*. The statistical significance has been estimated using the Benjamini-Hochberg Procedure, the asterisk indicates a significance of *p* < 0.05.

Based on the differential gene regulation observed in *B. bronchiseptica* we hypothesized that similar transcriptional changes would occur in *B. pertussis* and *B. parapertussis* after blood or serum exposure. *B. bronchispetica, B. pertussis*, and *B. parapertussis* were incubated in serum to study changes in gene expression because virulence genes were up-regulated in both, blood and serum, and it is easier to obtain RNA after serum incubation because blood was inhibiting the amplification process. Expression of the ACT gene *cyaA* and three genes of the T3SS (*bopN*, *bscO*, and *bsp22*), all of which were up-regulated in the microarray data (**Figure 1**), were measured using qRT-PCR. To validate the results obtained by qRT-PCR we compared those with the data obtained by microarray (*cyaA*: 5.33-fold w/2.34 stdev; *bopN*: 4.77-fold w/4.77 stdev; *bscO*: 8.9-fold w/7.47 stdev; and *bsp22*: 9.81-fold w/8.77 stdev up-regulated after 60 min serum exposure) in both cases two-fold increase was observed. Similar to *B. bronchiseptica*, expression of these genes was also significantly increased in *B. pertussis*. In *B. parapertussis* after serum exposure a significant increase was revealed for *bsp22* and *cyaA*, but not for *bopN* or *bscO* (**Figure 2**).

**FIGURE 3:**
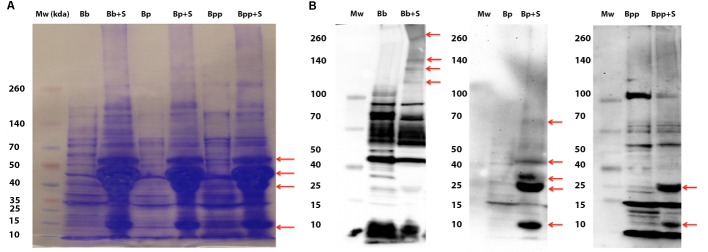
Exposure to serum induces differential protein production in the classical bordetellae. **(A)** Coomassie stained bacterial lysates from *B. bronchiseptica* strain RB50 (BB), *B. pertussis* strain 536 (BP), and *B. parapertussis* (BPP) strain 12822 from cultures incubated in SS medium or in 100% serum (+S). Red arrows indicate differentially produced proteins. **(B)** Western blot of the same lysates probed with mouse serum raised against each of the strains. Arrows indicate antigens induced by incubation in serum.

Polyacrylamide gel electrophoresis and western immuno-blot were performed on bacterial lysates to visualize differential protein and antigenic profile among the *Bordetella* spp. in response to serum exposure (**Figure 3**) (Cotter and Miller, 1997). Again, we incubated only with serum because it produced cleaner gels. We observed increased expression of similarly sized protein bands in all three species after exposure to serum. To assess whether the differential protein production affects expression of the various antigens believed to be important in infection and the subsequent immune recognition, Western Blots of bacterial lysates prepared from bacteria pre-incubated with sheep serum or not were generated from each of the three *Bordetella* subspecies. Each lysate was probed with serum, raised in mice infected with the three *Bordetella* species, respectively. All three blots displayed differences in the antigenic profile between lysates of bacteria exposed with and without naïve sheep serum (**Figure 3B** and **Supplementary Figure 1**). Interestingly, the antigenic profiles also differed among the three species. In *B. bronchiseptica*, exposure to serum induced production of high molecular weight antigens, but production of smaller antigens of approximately 25 and 12 kDa was induced in both *B. pertussis* and *B. parapertussis*. Albeit this is a crude assay as we used antibodies from a convalescent animal as primary antibody, it is a good indication that the expression of antigenic proteins varies after contact with host tissue. Together, these results indicate differential production of several antigens in response to exposure to naïve sheep serum (**Figure 3**). Interestingly, exposure to serum also induced the expression of antigens in the Bvg^−^ phase locked strain RB54, consistent with the previous data suggesting that not all BSRS are Bvg-dependent (**Supplementary Figure 1**). To ensure that there was no non-specific binding from the serum proteins, a Western Blot was performed using only serum. A coomassie stained gel shows a large amount of protein present in the serum, but the blot itself shows no false positives after incubation with serum of convalescent mice infected with *B. bronchiseptica, B. pertussis*, or *B. parapertussis* (**Supplementary Figure 2**).

### Exposure to Serum Increases Macrophage Cytotoxicity

**FIGURE 4:**
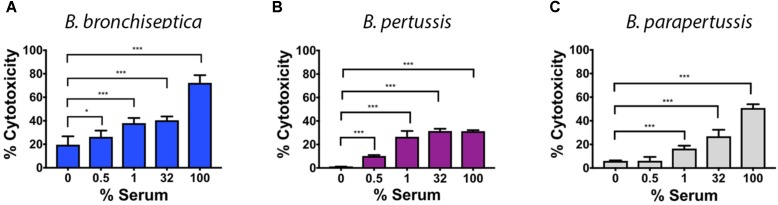
Serum enhances cytotoxicity of the classical bordetellae against macrophages. Cytotoxicity against macrophages was measured by LDH release of classical bordetellae incubated with increasing sheep serum concentrations against macrophages, measured as production of LDH. **(A)** Cytotoxicity of *B. bronchiseptica* against RAW 246.7 murine macrophages. **(B)** Cytotoxicity of *B. pertussis* against THP-1 human macrophages. **(C)** Cytotoxicity of *B. parapertussis* against THP-1 human macrophages. The asterisks indicate a significant difference with a *p*-value of *p* < 0.005 using the two-way ANOVA test.

Given the induced transcriptional changes and the altered antigenic profile after blood or serum exposure, we hypothesized that blood or serum exposure would also lead to an increase in virulence associated phenotypes such as cytotoxicity for phagocytic cells encountered in blood. Specifically, we expected the cytotoxicity to change in a manner that was dependent on serum concentration. We tested cytotoxicity following serum incubation because blood interferes with the colorimetric assay. To test this, we measured the cytotoxicity of *Bordetella* species pre-incubated with different serum concentrations. All three *Bordetella* species exhibited an increase in cytotoxicity toward macrophages in a dose dependent manner (**Figure 4**), however, the degree of cytotoxicity differed among species. While the average cytotoxicity of *B. bronchiseptica* increased from 25 to 68%, that of *B. pertussis* increased from 1 to 30%, and from 6 to 53% in *B. parapertussis*. To confirm that bacterial growth/death is not affecting the results, a control of only bacteria was performed. The results indicate that bacterial growth/death do not affect LDH measurements (**Supplementary Figure 3**). Increased cytotoxicity was also observed in *B. bronchiseptica* Bvg^+^ (65–90%) and Bvg^−^ (7–57%) phase-locked mutants, further supporting aspects of this response being Bvg^−^independent (**Supplementary Figure 4**).

These experiments were motivated by the hypothesis that *Bordetella* spp. respond to blood/serum as a signal of inflammation or invasion of deeper tissues, inducing expression of factors involved in resisting and/or modulating the considerable immune challenges there. We predict this response should be transient and highly conditional on the local microenvironment, and our results showing that Bvg status affects some, but not all, of the BSRS is evidence that there are complexities involved in optimizing regulation of virulence factors. Consistent with our expectation, we observed that pre-incubating *B. bronchiseptica* in serum or blood before inoculating animals did not affect the course of infection (**Supplementary Figures 5**, **6**). These results support the view that there is not simply an “on” switch for the BSRS; incubation in serum transiently turns on some virulence factors, but does not make the bacteria more virulent over the following week of infection.

## Discussion

Several studies have examined how bacteria adapt to host environments, including response to blood by pathogens such as *Staphylococcus aureus* (Malachowa and DeLeo, 2011; Malachowa et al., 2011), *Streptococcus* spp. (Graham et al., 2005; Mereghetti et al., 2008a,b), or *Enterococcus faecalis* (Solheim et al., 2007; Vebø et al., 2009). Here we show that the classical bordetellae, *B. bronchiseptica, B. pertussis*, and *B. parapertussis*, change their antigenic profiles in response to blood or serum exposure, and that genes comprising the BSRS are involved in iron metabolism, in transmembrane transport, and importantly in virulence.

Up-regulation of virulence factors such as ACT has important consequences for host–pathogen interactions. ACT plays a key role in manipulation of the host immune response by binding to the β2 integrin expressed on macrophages, neutrophils, dendritic cells, and natural killer cells (Ishibashi et al., 1994), and by inhibiting cytokine production, contributing to its role in manipulation of inflammation (Ross et al., 2004; Hickey et al., 2008; Henderson et al., 2012). Moreover, ACT production was increased in *B. pertussis* after exposure to serum, to calcium and to albumin (Gonyar et al., 2017), which led the authors to speculate that recognition of albumin may be the basic mechanism by which *B. pertussis* recognizes the host and establishes infection (Gonyar et al., 2017). This, together with our data, suggests that *Bordetella* spp. increase ACT production in response to sensing blood components to obtain nutrients and manipulate host immunity, increasing colonization of, and persistence in, the host.

The expression of the T3SS was increased in all three classical *Bordetella* species after exposure to serum. Up-regulation of T3SS has been previously reported under iron starvation (Brickman et al., 2011) or in CO_2_ levels approximating those in mammalian tissues (∼5%) (Hester et al., 2012). In *B. bronchiseptica*, the T3SS consists of approximately 30 proteins encoded by the *bsc* locus and regulated by several factors including the RNA-binding protein *Hfq* (Bibova et al., 2015), BB1618 (Kurushima et al., 2012), BvgAS (Nicholson et al., 2009), and BtrA/S (Ahuja et al., 2016). The T3SS plays a key role in colonization, (Nicholson et al., 2014) persistence and virulence (Skinner et al., 2005; Pilione and Harvill, 2006; Buboltz et al., 2009). The T3SS modulates the immune response by inducing apoptosis in leukocytes as well as by inactivation of NF-κB (Yuk et al., 2000). T3SS effector protein *BopN* induces IL-10 production in host cells, thereby inhibiting pro-inflammatory responses, which may allow persistent colonization (Nagamatsu et al., 2009). These results suggest that transcriptional activation of T3SS upon contact with blood components enables *Bordetella* spp. to manipulate the host’s immune response and facilitate persistence.

The cytotoxicity of *B. bronchiseptica* to macrophages is known to be Bvg-regulated, and the Bvg^+^ phase-locked mutant RB53 exhibited a considerably stronger cytotoxic effect (65% cytotoxicity) than the RB50 wild type (25%) and the Bvg^−^ phase locked mutant RB54 (7%). However, the cytotoxicity of all strains, including the Bvg^−^ phase locked strain RB54, intensified with increasing concentrations of sheep serum (**Figure 4**). This increased cytotoxicity might be the result of an increase expression of virulence factors such as ACT which increases cytotoxicity by two possible mechanisms, cAMP accumulation/ATP depletion and oligomerization/pore formation, each of which can be further modulated (Hewlett et al., 2006).

It is particularly intriguing to note that *Bordetella* spp. respond to blood and serum, potent signals of local inflammation, by changing their expression of molecules such as ACT and TTSS, each of which can affect local inflammation. The ability to finely control local inflammation would explain the remarkable success of these species in efficiently colonizing, spreading and persisting for life in some hosts, or, alternatively, inducing virulent coughing symptoms that facilitate rapid spread in others.

In summary, *Bordetella* spp. sense their host’s signals and differentially regulate gene expression to adapt to the host environment. Here we have identified a *Bordetella* Blood/Serum Responsive Stimulon (BSRS), which induces changes in the antigenic profile of the classical bordetellae, increases expression of several virulence factors and increases cytotoxicity. Further, we have shown that some of these responses are Bvg-dependent while others are Bvg^−^independent, further supporting the increasingly complicated view of how *Bordetella* species are able to regulate gene expression during infection.

## Author Contributions

MG, TN, and EH designed the experiments. MG, IR, LH, TN, KD, IHS, and MD conducted the experiments and acquired the data. MG, IR, TN, and KD analyzed the data. MG and BL wrote the manuscript. LH, BL, TN, KD, MD, and EH edited the manuscript. EH aquired the funding.

## Conflict of Interest Statement

The authors declare that the research was conducted in the absence of any commercial or financial relationships that could be construed as a potential conflict of interest.
