# Supplementary figures and images for: Blood or Serum Exposure Induce Global Transcriptional Changes, Altered Antigenic Profile, and Increased Cytotoxicity by Classical Bordetellae
Source: Front Microbiol. 2018 Sep 7;9:1969. doi: 10.3389/fmicb.2018.01969 (PMC6137168; doi:10.3389/fmicb.2018.01969)

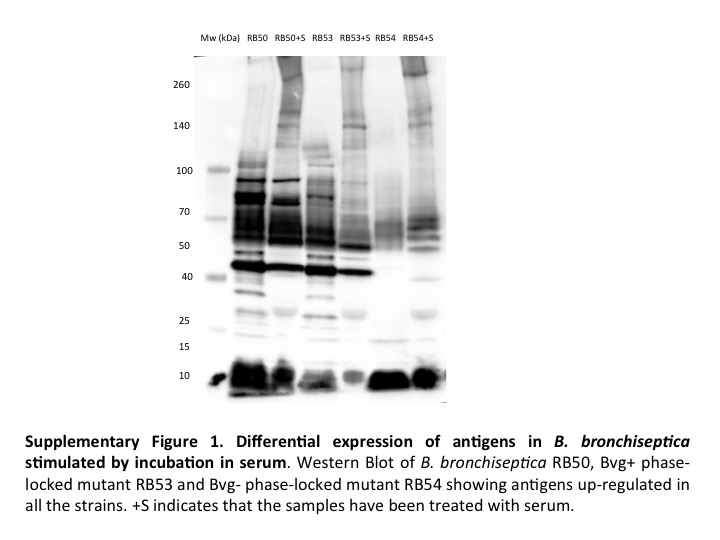

Supplement: Supplementary file 1 [file Image_1.JPEG]

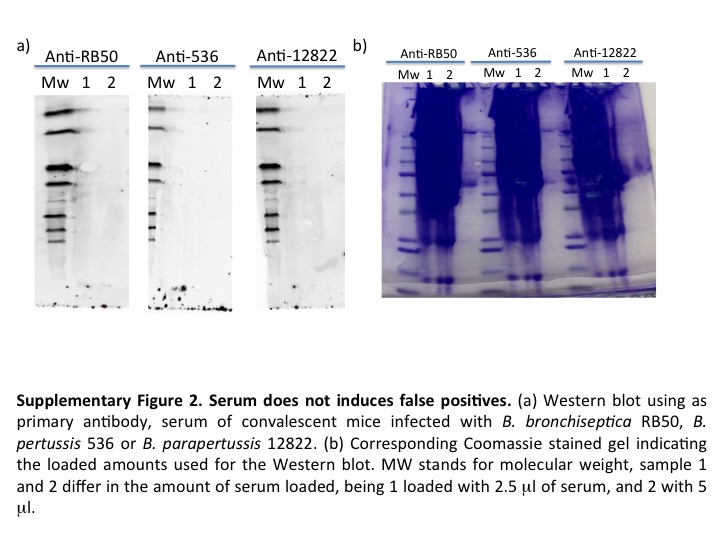

Supplement: Supplementary file 2 [file Image_2.JPEG]

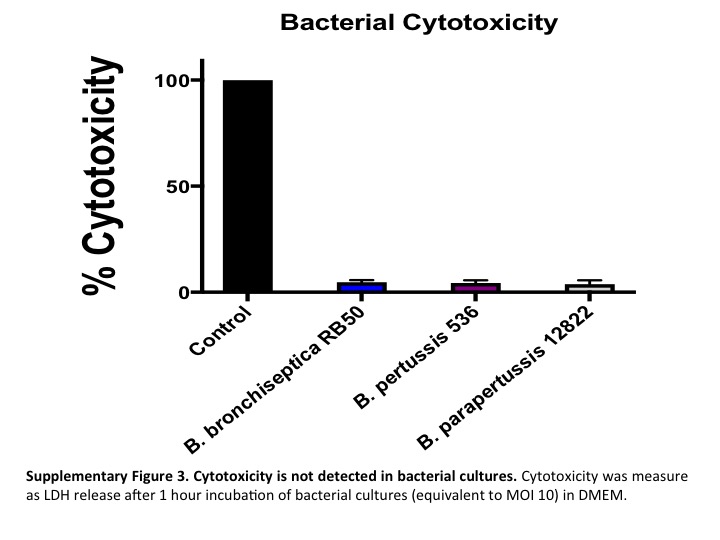

Supplement: Supplementary file 3 [file Image_3.JPEG]

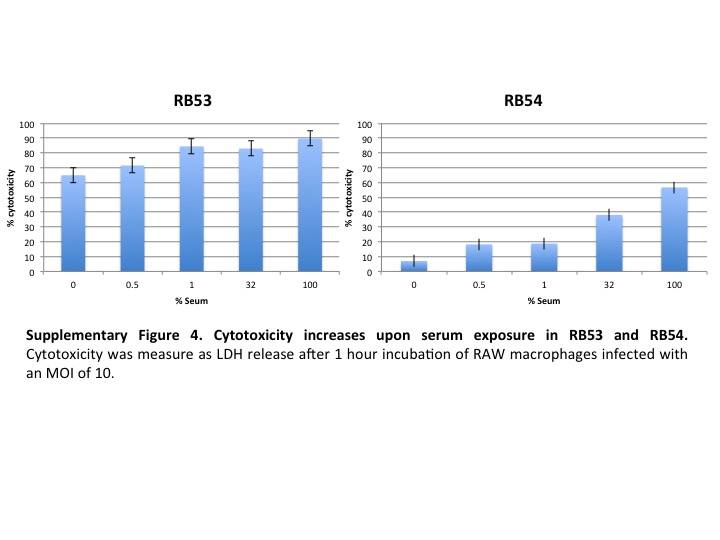

Supplement: Supplementary file 4 [file Image_4.JPEG]

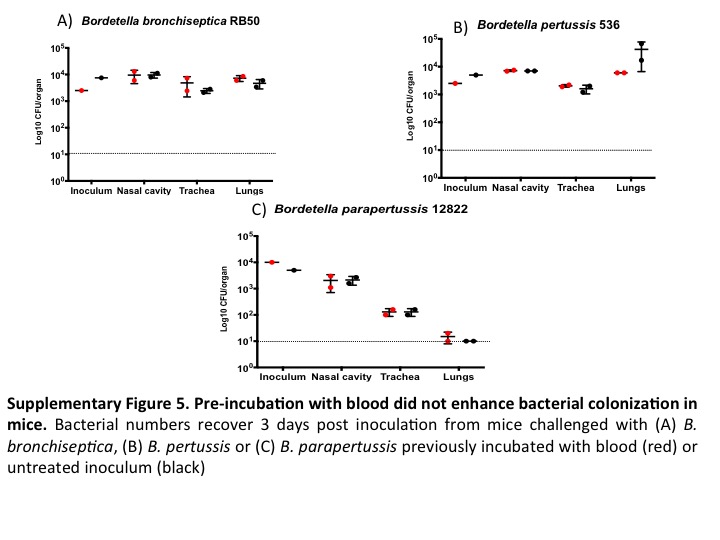

Supplement: Supplementary file 5 [file Image_5.JPEG]

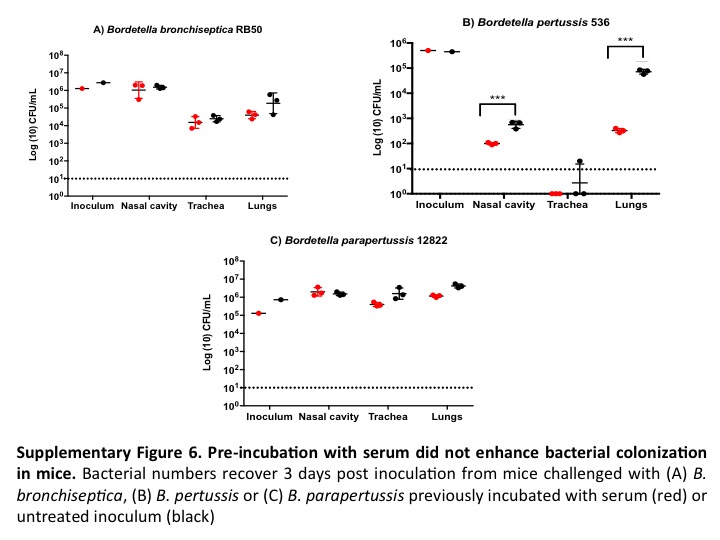

Supplement: Supplementary file 6 [file Image_6.JPEG]
