# Supplementary material for: Blood or Serum Exposure Induce Global Transcriptional Changes, Altered Antigenic Profile, and Increased Cytotoxicity by Classical Bordetellae
Source: Front Microbiol. 2018 Sep 7;9:1969. doi: 10.3389/fmicb.2018.01969 (PMC6137168; doi:10.3389/fmicb.2018.01969)
Supplement: Supplementary file 7 [file Table_1.DOCX]

**Supplementary table 1: Primers used in this study**

|  | **Forward** |  | **Reverse** |
| --- | --- | --- | --- |
| cyaA | CACTGAGCAGAACAATCCTTTCC | BB0324 | CGTGAGCATCTGGCTTTCAC |
| bopN | TGCCGAGGAAAAGCATCACT | BB1616 | GCCAGAGCATCGGACGTT |
| bsp22 | CGGCACGGGCGTCAT | BB1617 | GGTGTAGGCACTTTCGAGTTCCT |
| bscO | AGCTCGAAAACCGTCTTTATGC | BB1629 | TCGATGTCGCGTGTCTTGAC |
| BP_16S | TCAGCATGTCGCGGTGAAT | BP_16S | TGTGACGGGCGGTGTGTA |
| recA Fw | GCACCGAGGAATAGAACTTGAG | recA Rv | CAAGCTGACGGCCACCAT |
